# Magnitude of Anemia and Associated Factors among Pregnant Women Attending Antenatal Care in Public Hospitals of Ilu Abba Bora Zone, South West Ethiopia: A Cross-Sectional Study

**DOI:** 10.1155/2018/9201383

**Published:** 2018-11-12

**Authors:** Adamu Kenea, Efrem Negash, Lemi Bacha, Negash Wakgari

**Affiliations:** ^1^Faculty of Public Health and Medical Sciences, Mettu University, Mettu, Ethiopia; ^2^Department of Midwifery, College of Medicine and Health Sciences, Hawassa University, Hawassa, Ethiopia

## Abstract

**Background:**

Anemia is a global public health problem affecting all population particularly pregnant women. Hence, this study assessed the magnitude of anemia and associated factors among pregnant.

**Methods:**

Institution based cross-sectional study was conducted among 416 pregnant women attending antenatal clinic in three public hospitals of Ilu Aba Bora zone. The study participants were selected by proportional allocation based on the number of pregnant women that the respective health facilities contain. Semistructured questionnaire was used for data collection. Midupper arm circumference was employed to assess the nutritional status and standard mood depression assessment tool was used to assess depression. Data were centered and analyzed using SPSS version 20.0. Logistic regression analyses were used to see the association of different variables.

**Results:**

In this study, 31.5% of pregnant women were anemic. In addition, having family size five and above [AOR = 2.97, 95% CI (1.69, 5.27)], being rural resident [AOR=2.74, (95%CI) (2.11, 5.06)], had a higher odds of anemia. Similarly, having soil transmitted helminthes infection [AOR= 3.19, 95% CI (1.5, 6.65)] and history of malaria infection in the last one year [AOR= 3.10, 95% CI (2.10, 5.06)] had also a higher odds anemia during pregnancy. Moreover, being undernourished [AOR= 2.74 95% CI (1.34, 5.57)] was negatively associated with magnitude of anemia.

**Conclusions:**

The magnitude of anemia among pregnant women was found to be significant. Residence, family sizes, history of malaria infection during the last one year, and undernourishment were significantly associated with anemia during pregnancy.

## 1. Introduction

Anemia is global public health problem affecting people of different age groups. However, it is more prominent in pregnant women and young children and other reproductive age [1, 2]. According to the 2008 World Health Organization (WHO) report, anemia affected 1.62 billion (24.8%) people globally [3]. It had an estimated global prevalence of 42% in pregnant women and is a major cause of maternal mortality [4, 5] Sub-Saharan Africa is the most affected region, with anemia prevalence estimated to be 17.2 million among pregnant women, which corresponds to approximately 30% of total global cases [6].

Ethiopia is among countries where there is a high level of anemia among women of reproductive age (15-49years) and pregnant women. Seventeen percent of Ethiopian women age 15-49 are anemic, with thirteen percent having mild anemia, three percent having moderate anemia, and one percent having severe anemia [7]. A higher proportion of pregnant women are anemic (22%) than women who are breast feeding (19%) and women who are neither pregnant nor breastfeeding (15%) [8]. There is an increased iron requirement during pregnancy due to greater expansion in plasma volume that results in a decrease in hemoglobin (Hgb) level to 11g/dl [7]. Therefore, any Hgb level below 11g/dl in pregnancy is considered as anemia [7, 8]. Anemia could be classified as mild, moderate, and severe. The Hgb levels for each class of anemia in pregnancy are 10.0–10.9g/d1 (mild), 7–9.9g/dl (moderate), and <7g/dl (severe) [9].

The main risk factors for anemia are low intake of iron, poor absorption, high phytate, or phenolic compounds or increased requirements during childhood and pregnancy as well as infection with malaria, HIV, and hookworm [10–12]. The effect of anemia during pregnancy on maternal and neonatal life ranges from varying degrees of morbidity to mortality. As many studies reported, severe anemia (Hg < 7g/L) during pregnancy has been associated with major maternal and fetal complications. It increases the risk of preterm delivery [13, 14], low birth weight [13–16], intrauterine fetal death [16], neonatal death [17], maternal mortality [18], and infant mortality [19] associated with child hood intellectual disability [20, 21].

The availability of local information on the magnitude and related risk factors has a major role in the management and control of anemia in pregnancy. Even though the global and national prevalence of anemia among pregnant women and its associated factors were identified, it is not well determined in the study area. Therefore, this study intended to provide information about magnitude and factors that influenced anemia among pregnant women in south west Ethiopia and also used to assist: program planners and service providers in the study area to reduce maternal morbidity, mortality and serious complication of pregnancy or childbirth related to anemia.

## 2. Materials and Methods

### 2.1. Study Design, Setting and Population

Institution based quantitative cross-sectional study design was employed in public hospitals of Ilu Abba Bora zone. Ilu Abba Bora zone is found in south west Ethiopia. The zone is 600 km far away from Addis Ababa, capital of Ethiopia. The study conducted in three hospitals found in the zone; namely Mettu, Bedele and Darimu hospitals from January to July 2016. All pregnant women who were attending antenatal care in Ilu Abba Bora zone hospitals' ANC care unit were considered as source population. Those who are critically ill and unable to hear and/or speak are not long-term residents of the study area was excluded from the study.

### 2.2. Sample Size and Sampling Procedures

The sample size was calculated by using 56.7% prevalence of anemia among pregnant women attending antenatal clinic from a local study done in eastern Ethiopia [22]. Accordingly, the sample size for the study was calculated by using single proportion formula:

(1) $$\begin{array}{c} n=\frac{{\left(Z\alpha /2\right)}^{2}\times p\left(1-p\right)}{w^{2}} \end{array}$$

where n= sample size, p= prevalence of anemia, W = maximum allowable error = 0.05, and Z = value of standard normal distribution (Z-statistic) at 95% confidence level which is 1.96.Thus the computed sample size with 10% nonresponse rate became 416. The study subjects was selected by proportional allocation based on the number of pregnant women that the respective health facilities contain in their antenatal clinic and all pregnant women attending antenatal clinic during study period were included. Accordingly, from Mettu Karhl Hospital 176, Bedele Hospital 141, and Darimu Hospital l99 pregnant women were included.

### 2.3. Data Collection Instruments and Procedures

Semistructured interviewer administered questionnaire was employed to obtain data about sociodemographic characteristics, dietary intake and habit, gynecological factors, and patient-health care provider relationship. Document review was used to obtain medical condition of the mother (HIV and hemorrhoids). Midupper arm circumference measurement was used to assess the nutritional status of pregnant women while standard mood/depression assessment questionnaire was used to screen for depression. In addition, hemoglobin level was measured on site, using capillary blood samples collected using aseptic techniques, and intestinal parasites was checked by taking a single stool sample from each participant and history of malaria infection over the past one year was interviewed.

### 2.4. Data Quality Control

To ensure data quality, a pretested semistructured questionnaire and midupper arm circumference measuring instrument were used. Training about purpose of the study, how to approach study subjects, and how to use the questionnaire was given for three days for the data collectors and supervisors. The sample collections and laboratory procedure were done by senior experienced professionals. The collected data was checked for the completeness, accuracy, and clarity by the principal investigator and supervisors. This quality checking was done daily after data collection and correction was made before the next data collection measure. Data clean up and cross-checking were done before analysis.

### 2.5. Data Processing and Analysis

Data collected through a semistructured interviewer administered questionnaire was cleaned, coded, and entered into SPSS version 20.0 for analysis. Both bivariate and multivariate logistic regression analyses were used to determine the association of each independent variable with the dependent variable. Variables significant in bivariate analysis (p-value less than or equal to 0.2) were entered into a multivariate logistic regression model to adjust the effects of confounders on the outcome variable. Odds ratio with their 95% confidence intervals was computed to identify the presence and strength of association, and statistical significance was declared if p < 0.05.

### 2.6. Ethical Considerations

Ethical approval was obtained from the Research Ethical Review Committee at the Mettu University, Faculty of Public Health and Medical Sciences. Next, official letters were submitted to Oromia Regional Health Bureau. Then written permission was obtained from Oromia Regional Health Bureau and finally from Zonal Health office and respective health institution in the study area. During data collection process the data collectors informed each study participant about the purpose and anticipated benefits of the research project and the study participants. In addition they were informed about their full right to refuse, withdraw, or completely reject part or all of their part in the study and they were assured that their treatment and other benefits they gain from the hospitals will not be influenced by their participation in the study. Finally, they were asked for their informed written consent to participate or not to participate in the study and for their willingness on use of their files and records for the study.

## 3. Results

### 3.1. Sociodemographic Characteristics of the Respondents

A total of 416 pregnant women were involved in the study with a response rate of 100%. The mean age of the respondents was 25.15 (±4.29 years). Majority, 298 (71.7%), of them were urban dwellers while 118 (28.4%) of them were urban residents. More than two-thirds 144 (77.8%) of them had attended secondary education and more than half 223 (53.5%) of them were protestant religion followers. One hundred sixty-eight (40.4%) study participants have more than 1000 Ethiopian birr monthly income. The family sizes of the study participant were between 3 and 4 for 171 (41.1%) (Table 1).

### 3.2. Magnitude of Anemia

In this study the magnitude of anemia among pregnant women was found to be 31.5% [CI: 28.6-34.8]. The magnitude of anemia was high among respondents who were age between 39 and 45 (50%) and live in rural area 51(43.2%). Similarly, it was high among those with no formal education 47 (43.5%) and with family size five and above 42(39.3%). Moreover 28 (19.2%) of primi gravida and 84 (31.1%) of multi gravida women were anemic (Table 2). Among anemic pregnant women 69 (61.61%) of them had mild anemia, while 40 (35.7%) and 3(2.68%) of them had moderate and severe anemia, respectively.

### 3.3. Factors Associated with Magnitude of Anemia

In bivariate analysis the factors found to be significantly associated with magnitude of anaemia were age, residence, educational status, income, ethnicity, religion, marital status, delivery site, number of pregnancy, history of abortion, use of contraceptive, blood loss in the last delivery, malaria infection in the last one year, soil transmitted helminthes infection, HIV status, and nutritional status. However, residence, family size, soil transmitted helminthes infection, history of malaria infection in the last one year, and nutritional status remain significantly associated with magnitude of anaemia in the multivariate logistic regression. The odds of anemia among pregnant mothers who live in the rural area were 2.37 times higher than the odds of anemia among pregnant mothers who lives in the urban area [AOR=2.37, 95% CI (2.11, 5.06)]. Pregnant mothers with five and above family size were 2.97 times more likely to develop anemia as compared to pregnant mothers with less than five family sizes. Soil transmitted helminthes infection was significantly associated with anemia; pregnant mothers with soil transmitted helminthes infection were 3.19 times more likely to develop anemia than pregnant mothers without soil transmitted helminthes infection. In addition, history of malaria infection in the last one year [AOR= 3.10, 95% CI (2.10, 5.06)] had also a higher odds of anemia compared to their counterparts. Furthermore being undernourished had a higher odds of anemia compared well-nourished pregnant mothers [AOR= 2.74 95% CI (1.34, 5.57)] (Table 3).

## 4. Discussion

This study attempted to identify magnitude of anaemia among pregnant women attending antenatal clinic and associated factors in Ilu Aba Bora Zone, south west Ethiopia. The magnitude of anemia among pregnant women was found to be 31.5% [CI: 26.5-36.5]. With the reference to the WHO cutoff points [3], the present finding indicates moderate public health significance of anemia in the study area. This finding is higher than the studies conducted in other parts of Ethiopia, Awassa (15.3%) [23] and Gondar town (22%) [24]. Moreover, this finding is lower than the studies done in Gilegle Gibe (53.9%) [22] and west Arsi (36.6%) [25]. The possible reason for the observed difference might be due to difference in sociodemographic characteristics and geographical variation of the study participants which could be explained by the possible presence of food taboos during pregnancy in certain community in Ethiopia. For instance, the study conducted in southern Ethiopia indicated 65% of women avoided at least one food type during their recent pregnancy [26].

The presence of soil transmitted parasite infections was significantly associated with anemia in pregnant women (AOR=3.19, 95% CI = 1.50-6.65). This is consistent with study conducted in Jimma [27] and Gondar town [24]. This is because adult hookworm parasites attach and injure upper intestinal mucosa and also ingest blood. This brings about gastrointestinal blood loss and induces depletion of iron, folic acid, and vitamin B12 that ultimately lead to anemia [6]. Similarly, having history of malaria infection during the last one year also significantly associated with the magnitude of anemia during pregnancy (AOR= 3.10 95% CI (2.10, 5.06). This finding is consistent with other studies [13–15]. Malaria in pregnancy has been a cause of severe anemia. In areas with high prevalence of malaria, prevalence of anemia among pregnant women with malaria was up to 68.75% as compared to those without malaria infection (42.31%) [13]. Malaria causes hemolytic anemia and in severe cases it could also a risk factor for stillbirths, low birth weight, and fetal anemia [14, 15].

Place of residence become a factor for magnitude of anaemia during pregnancy. Pregnant mothers who lives in the rural area was 2.37 times more likely to develop anemia compared to who lives in the urban area [AOR=2.37, 95% CI (2.11, 5.06)]. This is in line with the study conducted in Sidama zone, southern Ethiopia [16]. In addition, those who have family size of five and above were 2.97 times likely to develop anemia as compared to pregnant mothers with less than five family sizes. Furthermore, this study revealed that anemia is 2.74 times more likely among under nourished pregnant women compared to well-nourished pregnant women [AOR=2.74, 95% CI (1.34, 5.57)]. This finding is consistent with the study done in Nigeria [18].

## 5. Conclusion

The magnitude of anemia among pregnant women was found to be significant among pregnant women. Rural residence, family size five and above, soil transmitted helminthes infection, and history of malaria infection during the last one year and the undernourished were significantly associated with anemia during pregnancy. To reduce the prevalence of anemia during pregnancy, there is a need to improve the frequency of dietary level, avoiding bare foot walk, constant use of insecticide treated bed net, and strength health care seeking behavior of women to ensure early diagnosis and management of soil transmitted helminthes and malaria, especially in rural areas.

Since information about factors associated with magnitude of anemia was obtained from respondents through interviewer administered questionnaires, rather than follow-up, response, recall, and social desirability bias are the potential limitations of this study. However, numerous scientific procedures have been employed to minimize the possible effects. For instance, procedures such as supervision, pretest of data collection tool, and adequate training of data collectors and supervisors were utilized.

## Figures and Tables

**Table 1:** Socio demographic characteristics of pregnant women attended antenatal clinic in public hospitals of Ilu Aba Bora zone, south west Ethiopia, 2016 (n=416).

**Variables**	**Yes[**%**]**	**No[**%**]**	**Total[**%**]**
**Age (years)**			
18-24	33[18.7]	143[81.25%]	176[42.3]
25-31	67[16.1]	143[68.1]	210[50.5]
32-38	10[35.7]	18[64.3]	28[6.7]
39-45	1[50]	1[50]	2[0.5]
**Educational status**			
No formal education	47[43.5]	61[56.5]	108[25.0]
Primary	20[27.7]	52[72.3]	72[17.3]
Secondary	32[22.2]	112[77.8]	144[34.6]
Higher education	13[14.1]	79[85.9]	92[22.1]
**Occupation **			
Farmer	41[51.3]	39[48.7]	80[19.2]
House wife	35[30.7]	79[69.3]	114[27.4]
Employee	28[16.4]	143[83.6]	171[41.1]
Others*∗*	9[17.6]	42[82.4]	51[12.3]
**Religion **			
Orthodox	39[31.7]	84[68.3]	123[29.6]
Muslim	17[28.8]	42[71.2]	59[14.2]
Protestant	55[24.8]	167[75.2]	222[53.4]
Catholic	2[16.7]	10[83.3]	12[2.9]
**Ethnicity **			
Amhara	4[50]	4[50]	9[1.9]
Tigre	7[28]	18[72]	25[6]
Oromo	101[26.4]	282[73.6]	383[92.1]
**Marital status **			
Married	100[25.2]	297[74.8]	397[95.4]
Others*∗∗*	12[41.4]	7[1.7]	19[4.6]
Monthly income			
<500	65[65]	35[35]	100[24]
500-1000	35[23.6]	113[76.4]	148[35.6]
>1000	12[7.1]	156[92.9]	168[40.4]
**Family size **			
<2	26[18.8]	112[81.2]	138[33.2]
3-4	44[25.7]	127[74.3]	171[41.1]
>5	42[39.3]	65[60.7]	107[27.7]

*∗*=Merchant, daily laborer *∗∗*=single, divorced, widowed

**Table 2:** The magnitude of anemia among pregnant women attended antenatal clinic in public hospitals of Ilu Aba Bora zone, south west Ethiopia, 2016 (n=416).

**Variables**	**Anemia**		**Total[**%**]**
	Yes[%]	No[%]	
**Gestational age **			
First trimester	12[32.4]	25[67.6]	37[8.9]
Second trimester	48[21.3]	177[78.7]	225[54.1]
Third trimester	52[33.8]	102[66.2]	154[37]
**Last delivery **			
Health institution	34[20.1]	135[79.9]	169[62.4]
Home	49[48.0]	53[52]	102[37.6]
**Birth interval **			
<2years	52[47.7]	57[52.2]	109[38.7]
>2years	36[20.8]	137[79.2]	173[61.3]
**Blood loss in last delivery**			
Yes	41[41.4]	58[58.6]	99[36]
No	44[25]	132[75]	176[64]
**History of abortion**			
Yes	38[49.4]	39[50.6]	77[18.5]
No	74[21.8]	265[78.2]	339[81.5]
**Use of contraceptive**			
Yes	48[22.1]	169[77.9]	217[52.2]
No	64[32.2]	135[67.8]	199[47.8]
**Malaria infection in the last one year**			
Yes	26[36.6]	45[63.4]	71[17.1]
No	86[24.9]	259[75.1]	345[82.9]
**STH infection** *∗*			
Yes	22[68.8]	10[31.2]	32[7.7]
No	90[23.4]	294[76.6]	384[92.3]
**HIV status**			
Positive	7[70]	3[30]	10[2.4]
Negative	105[25.9]	301[74.1]	406[97.6]
**Body mass index**			
Under weight	29[60.4]	19[39.6]	48[11.5]
Normal weight	77[25.3]	227[74.7]	304[73.1]
Over weight	6[9.4]	58[90.6]	64[15.4]

*∗*= Soil transmitted helminthes

**Table 3:** Factors associated with the magnitude of anemia among pregnant women visited antenatal clinic of public hospitals in Ilu Aba Bora zone, south west Ethiopia, 2016 (n=416).

**Variables**	**Anemia**		**OR(95**%** CI)**	
	**Yes[**%**]**	**No[**%**]**	**Crude OR**	**Adjusted OR**
**Residence**				
Urban	61[20.5]	237[39.5]		
Rural	51[43.2]	67[56.8]	2.96(1.62,6.92)	2.37(2.11,5.06)^*∗*^
**Marital status **				
Married	100[25.2]	297[74.8]	5.09(1.56,7.89)	1.03(0.87,1.76)
Others*∗∗*	12[41.4]	7[1.7]		
**Family size **				
<2	26[18.8]	112[81.2]		
3-4	44[25.7]	127[74.3]	1.49(0.46,7.65)	1.03(0.06,3.76)
>5	42[39.3]	65[60.7]	2.78(1.07,4.56)	2.97(1.69,5.27)^*∗*^
**Parity **				
Primi gravida	28[19.2]	118[80.8]		
Multi gravida	84[31.1]	186[68.9]	1.90(1.06,2.03)	1.61(0.54,2.43)
**Last delivery **				
Health institution	34[20.1]	135[79.9]	1.61(0.86,3.02)	1.28(0.54,3.01)
Home	49[48.0]	53[52]		
**Birth interval **				
<2years	52[47.7]	57[52.2]	1.15(0.56,2.38)	0.75(0.29,1.95)
>2years	36[20.8]	137[79.2]		
**Blood loss in last delivery**				
Yes	41[41.4]	58[58.6]	0.46(0.20,0.83)	0.45(.15, 1.29)
No	44[25]	132[75]		
**History of abortion**				
Yes	38[49.4]	39[50.6]		
No	74[21.8]	265[78.2]	0.41(0.20,0.83)	0.45(.15, 1.29)
**Use of contractive**				
Yes	48[22.1]	169[77.9]		0.58(0.251.365)
No	64[32.2]	135[67.8]		
**Malaria infection in the last 1 year**				
Yes	26[36.6]	45[63.4]	3.35(1.62,6.92)	3.1(2.1; 5.06)^*∗*^
No	86[24.9]	259[75.1]		
**Soil transmitted helminthes infection**				
Yes	22[68.8]	10[31.2]	3.2(1.6,6.32)*∗*	3.19(1.5,6.65)^*∗*^
No	90[23.4]	294[76.6]		
**HIV status**				
Positive	7[70]	3[30]	1.37(0.65,2.88)	1.15(0.47,2.83)
Negative	105[25.9]	301[74.1]		
**Body mass index**				
Under weight	29[60.4]	19[39.6]	4.49(1.56,5.63)*∗*	2.74(1.34,5.57)^*∗*^
Over weight	6[9.4]	58[90.6]	0.30(0.04,0.82)	0.96 (0.44,5.00)
Normal weight	77[25.3]	227[74.7]		

**∗** **=**significant in backward stepwise logistic regression and *∗∗*= single, divorced, and widowed.

## Data Availability

All data and materials in this manuscript could be deposited in publicly available repositories.
